# Supplementary material for: Comparison between radiofrequency ablation and sublobar resections for the therapy of stage I non-small cell lung cancer: a meta-analysis
Source: PeerJ. 2020 May 21;8:e9228. doi: 10.7717/peerj.9228 (PMC7246024; doi:10.7717/peerj.9228)
Supplement: Supplemental Information 1 [file peerj-08-9228-s001.doc]

| **Section/topic** | **#** | **Checklist item** | **Reported on page #** |
| --- | --- | --- | --- |
| **TITLE** | | |  |
| Title | 1 | Identify the report as a systematic review, meta-analysis, or both. | 1 |
| **ABSTRACT** | | |  |
| Structured summary | 2 | Provide a structured summary including, as applicable: background; objectives; data sources; study eligibility criteria, participants, and interventions; study appraisal and synthesis methods; results; limitations; conclusions and implications of key findings; systematic review registration number. | 2-3 |
| **INTRODUCTION** | | |  |
| Rationale | 3 | Describe the rationale for the review in the context of what is already known. | 5 |
| Objectives | 4 | Provide an explicit statement of questions being addressed with reference to participants, interventions, comparisons, outcomes, and study design (PICOS). | 5 |
| **METHODS** | | |  |
| Protocol and registration | 5 | Indicate if a review protocol exists, if and where it can be accessed (e.g., Web address), and, if available, provide registration information including registration number. | 5-6 |
| Eligibility criteria | 6 | Specify study characteristics (e.g., PICOS, length of follow-up) and report characteristics (e.g., years considered, language, publication status) used as criteria for eligibility, giving rationale. | 6-7 |
| Information sources | 7 | Describe all information sources (e.g., databases with dates of coverage, contact with study authors to identify additional studies) in the search and date last searched. | 6 |
| Search | 8 | Present full electronic search strategy for at least one database, including any limits used, such that it could be repeated. | 6 |
| Study selection | 9 | State the process for selecting studies (i.e., screening, eligibility, included in systematic review, and, if applicable, included in the meta-analysis). | 6-7 |
| Data collection process | 10 | Describe method of data extraction from reports (e.g., piloted forms, independently, in duplicate) and any processes for obtaining and confirming data from investigators. | 7-8 |
| Data items | 11 | List and define all variables for which data were sought (e.g., PICOS, funding sources) and any assumptions and simplifications made. | 4-5,8 |
| Risk of bias in individual studies | 12 | Describe methods used for assessing risk of bias of individual studies (including specification of whether this was done at the study or outcome level), and how this information is to be used in any data synthesis. | None |
| Summary measures | 13 | State the principal summary measures (e.g., risk ratio, difference in means). | 8 |
| Synthesis of results | 14 | Describe the methods of handling data and combining results of studies, if done, including measures of consistency (e.g., I^2^) for each meta-analysis. | 8-9 |

Page 1 of 2

| **Section/topic** | **#** | **Checklist item** | **Reported on page #** |
| --- | --- | --- | --- |
| Risk of bias across studies | 15 | Specify any assessment of risk of bias that may affect the cumulative evidence (e.g., publication bias, selective reporting within studies). | 10 |
| Additional analyses | 16 | Describe methods of additional analyses (e.g., sensitivity or subgroup analyses, meta-regression), if done, indicating which were pre-specified. | None |
| **RESULTS** | | |  |
| Study selection | 17 | Give numbers of studies screened, assessed for eligibility, and included in the review, with reasons for exclusions at each stage, ideally with a flow diagram. | 9 |
| Study characteristics | 18 | For each study, present characteristics for which data were extracted (e.g., study size, PICOS, follow-up period) and provide the citations. | 9 |
| Risk of bias within studies | 19 | Present data on risk of bias of each study and, if available, any outcome level assessment (see item 12). | None |
| Results of individual studies | 20 | For all outcomes considered (benefits or harms), present, for each study: (a) simple summary data for each intervention group (b) effect estimates and confidence intervals, ideally with a forest plot. | 11 |
| Synthesis of results | 21 | Present results of each meta-analysis done, including confidence intervals and measures of consistency. | 10-11 |
| Risk of bias across studies | 22 | Present results of any assessment of risk of bias across studies (see Item 15). | 12 |
| Additional analysis | 23 | Give results of additional analyses, if done (e.g., sensitivity or subgroup analyses, meta-regression [see Item 16]). | None |
| **DISCUSSION** | | |  |
| Summary of evidence | 24 | Summarize the main findings including the strength of evidence for each main outcome; consider their relevance to key groups (e.g., healthcare providers, users, and policy makers). | 13-15 |
| Limitations | 25 | Discuss limitations at study and outcome level (e.g., risk of bias), and at review-level (e.g., incomplete retrieval of identified research, reporting bias). | 17-18 |
| Conclusions | 26 | Provide a general interpretation of the results in the context of other evidence, and implications for future research. | 18 |
| **FUNDING** | | |  |
| Funding | 27 | Describe sources of funding for the systematic review and other support (e.g., supply of data); role of funders for the systematic review. | 18-19 |

*From:*  Moher D, Liberati A, Tetzlaff J, Altman DG, The PRISMA Group (2009). Preferred Reporting Items for Systematic Reviews and Meta-Analyses: The PRISMA Statement. PLoS Med 6(7): e1000097. doi:10.1371/journal.pmed1000097

For more information, visit: **www.prisma-statement.org**.

Page 2 of 2

**Title：**

Comparison between Radiofrequency Ablation and Sub-Lobar Resections for the Therapy of Stage I Non-Small Cell Lung Cancer: A Meta-Analysis

**Structured summary：**

Background. Sub-lobar resection (SLR) and radiofrequency ablation (RFA) are the two minimally invasive procedures performed for treating stage I non-small cell lung cancer(NSCLC). This study aims to compare SLR and RFA for the treatment of stage I NSCLC using the meta-analytical method.

Methods. We searched PubMed and Embase for articles published till December 2019 to evaluate the comparative studies assessing the survival rates, progression-free survival rates, and post-operative complications (PROSPERO registration number: 176765). A meta-analysis was performed by combining the outcomes of the reported incidence of short-term morbidity and long-term mortality. The fixed or random effects model was utilized to calculate the pooled odds ratios(ORs) and the 95% confidence intervals.

Results. A total of four retrospective studies were considered in the course of this study. The studies included comprised 309 participants, 154 participants were assigned to the SLR group, and 155 participants were assigned to the RFA group. Moreover, there were statistically significant differences between the one- and three-year survival rates and one- and three-year progression-free survival rates which favored SLR, between the two groups. Postoperative complications, including pneumothorax and pleural effusion were in favor of SLR, except for cardiac abnormality. A difference between SLR and RFA with respect to hemoptysis was not observed, which was possibly attributed to the limited sample size in this study.

Conclusion: Considering the higher survival rates and disease control in cases of stage I NSCLC, surgical resection is the more preferred method of treatment compared to RFA. RFA can be considered a valid alternative for patients ineligible for operation and high-risk patients due to the shorter its short hospital stay and less low invasiveness associated with RFA.

**Rationale：**

The superiority of SLR over RFA is still controversial.

**Objectives:**

This study aimed to evaluate the postoperative complications and survival rates of patients with stage I NSCLC who underwent SLR or RFA.

**Protocol and registration**

Our research was registered on PROSPERO registration (176765). Electronic searches were conducted by two investigators (Shuang Chen and Sheze Yang) using the PubMed, Cochrane Library, Web of Science, Ovid MEDILINE, Google Scholar and Embase databases until December 2019.

**Eligibility criteria**

(1) Studies that compared the survival situation and post-operative complication rates of SLR and RFA in patients with stage I NSCLC

(2) Articles that contained the survival data of reports that were presented at major radiology and thoracic surgery academic conferences (RSNA, AATS and EACTS) or studies that were published in peer-reviewed publications

(3) Studies comprising participants with similar main clinical characteristics and with no history of malignant tumors

(4) Studies that reported at least one of the following results: survival, progression-free survival, and local recurrence rates(full texts were retrieved from studies that met all the inclusion criteria, and SLR refers to wedge resection and segmentectomy)

**Information sources**

Electronic searches were conducted by two investigators using the PubMed, Cochrane Library, Web of Science, Ovid MEDILINE, Google Scholar and Embase databases until December 2019.

**Search**

The following search Medical Subject Heading terms and keywords were included in our search strategy in a variety of combinations: “radiofrequency ablation,” “sub-lobar resections,” “non-small cell lung cancer,” “stage I,” “wedge resection,” “segmentectomy,” “RFA,” and “SLR.”

**Study selection**

Randomized intervention studies or observational cohort researches were eligible for inclusion if they followed up patients for at least 3 years, and the predefined inclusion criteria were as follows:

(1) Studies that compared the survival situation and post-operative complication rates of SLR and RFA in patients with stage I NSCLC

(2) Articles that contained the survival data of reports that were presented at major radiology and thoracic surgery academic conferences (RSNA, AATS and EACTS) or studies that were published in peer-reviewed publications

(3) Studies comprising participants with similar main clinical characteristics and with no history of malignant tumors

(4) Studies that reported at least one of the following results: survival, progression-free survival, and local recurrence rates(full texts were retrieved from studies that met all the inclusion criteria, and SLR refers to wedge resection and segmentectomy)

The following studies were excluded:

(1) Articles that did not comprise a comparison group with SLR or RFA as a method of interventions and studies evaluating stage II, III, and IV NSCLC;

(2) Researches focusing on participants undergoing treatment for pulmonary metastases

(3) Published articles with an overlap among researchers, hospitals, institutions, or participant cohorts(only the most informative and newest literature was adapted)

(4) Articles that were published for the past 20 years considering the recent remarkable technological advancements

**Data collection process**

All the related studies were searched and reviewed by two independent investigators (Shuang Chen and Shize Yang) according to our predefined eligibility criteria. The investigators also extracted research data, study design, and baseline characteristics (age and sex) and endpoints according to the predesigned data extraction form. Any needed but incomplete survival information in the articles was acquired by directly contacting the author. Different data extraction results among the two investigators were solved through discussion by both parties and finally overseen by a third senior independent author (Siyuan Dong). The final extracted data were confirmed by two senior investigators (Siyuan Dong and Shun Xu). All the included studies were assessed for quality based on the Downs and Black quality assessment method used in our previous studies(Dong et al. 2015; Dong et al. 2014; Downs & Black 1998). The progression-free survival was defined as the period between the date of the initial surgical resection or RFA until the date of a recurrence.

**Data items**

SLR, which is also referred to as limited resection, is preferred for patients who cannot tolerate lobectomy with systematic mediastinal lymphadenectomy specifically to preserve pulmonary function. SLR can be performed by anatomical segmentectomy or non-anatomical wedge resection through an open thoracotomy or with a video-assisted thoracoscopic surgery (VATS) approach. RFA is a minimally invasive approach that utilizes CT-guided percutaneous placement of an electrode into the lesion and generation of high-dose energy to cause coagulation necrosis. The progression-free survival was defined as the period between the date of the initial surgical resection or RFA until the date of a recurrence.

**Summary measures**

Survival data are reported as hazard ratio and dichotomous clinical outcomes as risk ratio. The corresponding 95% confidence interval (95% CI) was calculated. Moreover, a p value <0.05 was considered statistically significant when assessing the value between SLR and RFA.

**Synthesis of results**

A fixed effects model was adapted, if there was no statistically significant difference in terms of heterogeneity (p>0.05). Otherwise, a random effect model was adopted. Heterogeneity between all the included articles was investigated using the I2 statistic with statistical significance P<0.05. Hence, the upper thresholds for low, moderate, and high heterogeneity were defined as I2 values between 25% and 50%, between 50% and 75%, and greater than 75%, respectively.

**Risk of bias across studies**

Visual inspection of funnel plots was applied to assess the potential publication bias.

**Study selection**

Four retrospective cohort studies that met our inclusion criteria were adapted in our study between 2010 and 2015: two from the USA and two from Germany and Italy. A total of 309 participants were included in these researches: 154 participants were assigned to the SLR group, and 155 participants were assigned to the RFA group to assess the postoperative complications and survival rates.

**Study characteristics**

All the included articles’ basic characteristics and evaluation index are presented in Table 1. Alexander’s(Alexander et al. 2013), Ambrogi’s(Ambrogi et al. 2015), and Safi’s(Safi et al. 2015) researches were in favor of SLR; however, in Zemlyak’s(Zemlyak et al. 2010) study, RFA demonstrated a comparable effect to that of SLR in participants with stage I NSCLC.

**Results of individual studies**

All the survival rate results are presented in Table 2.

**Synthesis of results**

All four studies reported the results of 1-year survival rate(Alexander et al. 2013; Ambrogi et al. 2015; Safi et al. 2015; Zemlyak et al. 2010), and no significant heterogeneity between the four researches was observed (x2=2.15, p=0.54, I2=0%). Thus, a fixed effects model was adapted (OR=3.34; 95% CI, 1.13-9.89; p=0.03, Figure 3 A). These four studies also reported the outcomes of 3-year survival rate, and heterogeneity was calculated through these researches (x2=4.18, p=0.24, I2=28%); and a fixed effects model was used (OR=1.95; 95% CI, 1.20–3.18; p = 0.007, Figure 3 B). Only one paper(Ambrogi et al. 2015) documented the results of 5-year survival rate(52% for SLR and 35% for RFA); hence, the results could not be synthesized.. All the results were in favor of SLR.

Three articles(Ambrogi et al. 2015; Safi et al. 2015; Zemlyak et al. 2010) compared the 1-year progression-free survival rate (OR=2.72; 95% CI, 1.18–6.29; p=0.02), and no significant heterogeneity among these articles was detected (x2=0.34, p=0.84, I2=0%, Figure 4A). All three studies continued to reveal the 3-year progression-free survival rate (OR=3.01; 95%CI, 1.63–5.55; p=0.0004), and a significant heterogeneity among the participants who received SLR and those who received RFA was not observed (x2=2.00, p=0.37, I2=0%, Figure 4B). Significant 1- and 3-year progression-free survival rate benefits with SLR were observed. We also intended to compare the 5-year progression-free survival rate result between the two groups.

**Risk of bias across studies**

Publication bias is possibly observed when non-significant outcomes remain unpublished, inevitably amplifying the evident magnitude of a function. This research’s funnel plots are demonstrated in Figure 5. The funnel plots of the 1-year survival rate following SLR and RFA for the treatment of stage I NSCLC manifested a slight asymmetry, and all points were within 95% CI, suggesting a low publication bias.

**Summary of evidence**

RFA is a percutaneous treatment performed with local anesthesia and conscious sedation(Choe et al. 2009; Palussiere et al. 2015). Individuals who highly favor RFA emphasize that the procedure presents some definitive advantages over surgery, including treatment in an outpatient setting and the percutaneous performance of this procedure using of local anesthesia, avoiding a thoracotomy for patients who refuse surgical resection or with severe co-morbidities. RFA does not significantly affect the patient’s cardio-pulmonary function, and it is also associated with a significant decrease in hospital’s length of stay. Moreover, RFA allows the ablation of lesions without a major damage to the peripheral normal tissues(Simon et al. 2007; Wan et al. 2016a; Wan et al. 2016b). RFA complications are relatively minor and acute, although these complications are frequently experienced by patients. Zemlyak’s study(Zemlyak et al. 2010) showed that most patients undergoing RFA could be discharged within 24 hours of the therapy, and RFA has similar overall and cancer-specific survival rates to that of SLR. Considering that RFA does not lead to any loss of pulmonary function and that it can be repeatedly performed if a patient has a tumor recurrence or a new tumor growth, it also has the following several advantages: it is well tolerated by outpatients and is complementary to chemotherapy, treats of metachronous and synchronous lesions, results in rapid recovery of physical performance, and has a relatively short treatment time(Cheng et al. 2016; Chua et al. 2010; Li et al. 2013; Ridge et al. 2014). The following four complications were documented in our included research: hemoptysis, pneumothorax, pleural effusion, and cardiac abnormality. The most common complication is pneumothorax, and it was observed in every research and even more noted in the RFA group compared to the SLR group (Figure 2). Although the RFA group had more complications compared to the SLR group, the complications of RFA were relatively minor.

However, the following disadvantage is notably observed in RFA: the application of RFA is limited considering the close proximity of the vascular structure to the location and size of the tumor. The energy generated to the tumor will be reduced if the vessel’s diameter greater than 0.3cm due to the loss of energy through convection within the surrounding circulatory system(Lencioni et al. 2004). The proximity of the tumor to the trachea, heart, and esophagus increases the risk when performing RFA. Heat is also reduced if the tumor is greater than 3cm in diameter considering its periphery; hence, it is difficult to reach an ideal ablative temperature, resulting in an impaired local control and diminished response(Sharma et al. 2012). SLR has the following advantage: it has better oncologic outcomes compared to RFA. According to Keenan’s research(Keenan et al. 2004) comprising 201 stage I NSCLC patients, SLR has the following advantages: it better preserves lung function compared to lobectomy, and the forced vital capacity and forced expiratory volume in 1 second are better preserved in the SLR group than in the RFA group. Moreover, Kodama’s(Kodama et al. 1997) and Kates’s(Kates et al. 2011) studies both reveal that for T1N0 NSCLC, the survival rate of SLR is also similar to that of lobectomy.

Recently, several authors reported different VATS techniques to localize lung tumors, avoiding the necessity of open thoracotomy, which can be performed through a minimally invasive video-assisted thoracoscopic surgery(Ambrogi et al. 2012; Gruber-Rouh et al. 2017; Lin et al. 2016; Refai et al. 2020). Furthermore, an incidence rate of 5% of lymph node involvement was observed in 100 NSCLC patients with tumor <1cm in diameter, suggesting that nodal assessment should be taken into consideration even in small lesions(El-Sherif et al. 2005). Hence, SLR has the following additional advantage: in SLR, lymph node sampling can be performed at the time of surgical resection, allowing the identification of potential metastatic node and a more precise staging to guide treatment. Surgery has the following main disadvantage: not every patient can tolerate resection secondary to comorbid disease or poor reserve. Additionally, the SLR significantly requires a longer post-procedure length of stay and higher cost than RFA(Alexander et al. 2013).

**Limitations**

Despite the outcomes of our study, there are some limitations of this study. First, randomized controlled trials comparing RFA with SLR have not been conducted. Most available studies are single institution case series and small observational studies. Second, due to the retrospective design of the included studies, the patients undergoing RFA were older and had higher co-morbidity scores and lower performance compared with patients undergoing SLR; hence, patients undergoing RFA were categorically likely to die sooner considering that they were ineligible for operation. Although all the included studies focused on stage I NSCLC, the different stages of NSCLC also influence the patients’ survival and local recurrence. Third, the included studies exclusively focused on stage I NSCLC. It is known that the therapeutic effect of RFA is also closely associated with tumor size, but studies assessing the association between RFA and tumor size have not been conducted to date. Finally, the mediastinal and hilar lymph nodes are evaluated by preoperative imaging and not by pathologic verification when RFA is used. Hence, these real staging status of the patients may be underestimated, resulting in survival bias. Moreover, with RFA, the tumor is not eliminated, and a residual scar, which is commonly noted after the treatment, can be mistaken for a recrudescent lesion. The inclusion of additional RCTs among the studies we evaluated in this study would have increased the significance of our results.

**Conclusions**

In summary, the outcomes of our study, affirm that surgical intervention, even if limited to SLR, has a better survival rate compared with RFA and should therefore be performed whenever possible, excluding only patients ineligible for operation. Further large-scale prospective randomized studies are required not only to clearly compare the survival rates of different approaches, but also to better define the participants considered at high risk.

**Funding**

This research was supported by a grant (no. 81702242) from the National Science Foundation of China.
