# Supplementary material for: Comparison between radiofrequency ablation and sublobar resections for the therapy of stage I non-small cell lung cancer: a meta-analysis
Source: PeerJ. 2020 May 21;8:e9228. doi: 10.7717/peerj.9228 (PMC7246024; doi:10.7717/peerj.9228)
Supplement: Supplemental Information 2 [file peerj-08-9228-s002.docx]

**1 The rationale for conducting the meta-analysis.**

Lung cancer is still the leading cause of cancer deaths worldwide, with an estimated overall 5-year survival of 16 %. With development in imaging technology, many more early-stage lung cancers are being diagnosed than in the past, which may achieve a better long-term outcome. At present, the standard surgical treatment for localized stage I Non-small cell lung cancer (NSCLC) is lobectomy with systematic mediastinal lymphadenectomy. Though, surgical treatment provides patients with the best chance of total cure and long-term survival，resection is possible only in twenty percent of the cases. Patients which are candidates for lobectomy are often predisposed to advanced age, atherosclerotic cardiovascular disease, pulmonary dysfunction by cigarette smoking or other comorbidities.

Thus, less invasive modalities are being applied for these patients, such as sub-lobar resection(SLR), radiofrequency ablation(RFA) and stereotactic body radiation therapy (SBRT).

For those patients who are not suitable for lobectomy, SLR is recommended over nonsurgical therapy. However, the superiority of SLR over RFA is still controversial. To date, there is no proof from randomized controlled trial regarding the effect of RFA compared with SLR in stage I non-small cell lung cancer. With the aim of exploring whether RFA can get the survival comparable to that by SLR to stage I non-small cell lung cancer patients, we performed this research based on patients with stage I non-small lung cancer who underwent SLR or RFA to evaluate both the postoperative complication and survival rates.

**2 The contribution that the meta-analysis makes to knowledge in light of previously published related reports, including other meta-analyses and systematic reviews.**

This is the first meta-analysis for comparison between SLR and RFA for stage I non-small cell lung cancer. We assessed the clinical results of patients of stage I non-small cell lung cancer including five studies, comparing the survival rates by sub-lobar resection with those by CT-guided thermal ablation.
